# An antidiabetic nutraceutical combination of red yeast rice (*Monascus purpureus*), bitter gourd (*Momordica charantia*), and chromium alleviates dedifferentiation of pancreatic β cells in db/db mice

**DOI:** 10.1002/fsn3.1966

**Published:** 2020-10-25

**Authors:** Ke‐Ying Lu, Szu‐Han Chen, Yu‐Shun Lin, Hai‐Ping Wu, Pei‐Min Chao

**Affiliations:** ^1^ Department of Nutrition China Medical University Taichung Taiwan; ^2^ Lishui Municipal Central Hospital Lishui China

**Keywords:** β cell dedifferentiation, chromium, db/db mice, *Momordica charantia*, *Monascus purpureus*, type 2 diabetes

## Abstract

Antidiabetic properties of red yeast rice, bitter gourd, and chromium have gained scientific support. This study aimed to test whether a nutraceutical combination of these 3 materials prevented dedifferentiation of pancreatic β cells. Male db/db mice (8 weeks of age) were allocated into four groups (DB, DB/L, DB/M, and DB/H; *n* = 8–10) and fed a high‐fat diet containing 0%, 0.2%, 0.4%, or 1% nutraceutical, respectively, whereas wild‐type mice receiving a standard diet served as a healthy control (C; *n* = 10). The nutraceutical contained 10 mg/g monacolin K, 165 µg/g chromium, and 300 mg/g bitter gourd. After 8‐weeks dietary treatment, diabetic syndromes (including hyperglycemia, hyperphagia, excessive drinking, polyuria, glucosuria, albuminuria, and glucose intolerance), were improved by the nutraceutical in a dose‐dependent fashion. Decreased insulin and increased glucagon in serum and pancreatic islets in db/db mice were abolished in the DB/H group. Furthermore, supplementation curtailed dedifferentiation of β cells, as evidenced by decreasing the dedifferentiation marker (*Aldh1a3*) and increasing β‐cell‐enriched genes and transcription factors (*Ins1*, *Ins2*, FOXO1, and NKX6.1), as well as nuclear localization of NKX6.1 in pancreatic islets when compared to the DB group. We concluded that this nutraceutical, a combination of *Monascus purpureus*, *Momordica charantia*, and chromium, could be used as an adjunct for type 2 diabetes treatment and delay disease progression by sustaining β‐cell function.

## Graphical abstract

Beta‐cell dedifferentiation is an important issue leads to beta‐cell failure in type 2 diabetes. This study shows an antidiabetic nutraceutical combination of red yeast rice, bitter gourd, and chromium is able to curb beta‐cell dedifferentiation by decreasing the dedifferentiation marker (Aldh1a3) and increasing beta‐cell‐enriched genes (Ins1, Ins2, FOXO1, and NKX6.1), as well as nuclear localization of NKX6.1.

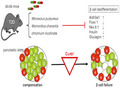

## 1 INTRODUCTION

Pathogenesis of type 2 diabetes (T2D) includes 2 essential components: insulin resistance and β‐cell dysfunction. Recently, the importance of the latter (reduced β‐cell function and/or mass) was emphasized, as it antedates and predicts disease onset and progress (Halban et al., 2014; Kahn, 2001). In response to insults that are exogenous (environment) or endogenous (genetic susceptibility), impairments of islet response involve mechanisms such as glucolipotoxicity, lipotoxicity, oxidative stress, endoplasmic reticulum stress, and inflammation (Halban et al., 2014), ultimately lead to β‐cell failure, a chronically progressive and partially reversible process. In contrast to β‐cell death or apoptosis, which is irreversible and maybe overestimated in patients (Marselli et al., 2014), β‐cell dedifferentiation occurs in T2D, with restoration possible at an early stage (Cinti et al., 2016; John et al., 2018; Talchai et al., 2012; Wang et al., 2017). Based on murine studies, curbing progress of β‐cell dedifferentiation (i.e., preserving functional β cells) is a successful antidiabetes strategy (Boland et al., 2019; Gómez‐Banoy et al., 2019; Ishida et al., 2017).

Foxkhead box protein O1 (FOXO1), a β‐cell‐enriched gene, protects β‐cell fate under metabolic stress. Upon acute demands, dephosphorylated FOXO1 is acetylated and translocated to the nucleus to compensate for insulin secretion by augmenting DNA transcription. However, ongoing stress causes increased ubiquitin‐dependent degradation of FOXO1 and consequently β‐cell exhaustion (Kitamura et al., 2005). Ablation of *Foxo1* in β cells causes hyperglycemia in multiparous or aged mice by downregulating expression of insulin and key β‐cell transcription factors, including the NK class of homeodomain‐encoding gene 6.1 (NKX6.1), pancreatic and duodenal homeobox 1 (PDX1), and MAF BZIP transcription factor A (MAFA) genes. Furthermore, genes ordinarily silenced in mature β cells, for example, pro‐endocrine and multipotency markers, α or δ cell hormone (glucagon or somatostatin), were detected in these dedifferentiated β cells (Talchai et al., 2012). These characteristics of β‐cell dedifferentiation, that is, loss of β‐cell identity along with reversion to an uncommitted endocrine progenitor stage or conversion into other endocrine cell types, were supported by RNA sequencing data of islets from wild‐type, db/+, and db/db mice (John et al., 2018) and immunohistology of human islets from diabetic‐ and nondiabetic organ donors (Cinti et al., 2016).

There are many medicinal foods with antidiabetic potential. *Monascus* fungus or red yeast is used as an ancient fermentative food in eastern Asia. In addition to conferring protection from dyslipidemia and cardiovascular diseases due to its famous monacolin (statin) metabolites, the antidiabetic potential of *Monascuc*‐fermented products was evident in animal studies (Lin et al., 2017; Shi & Pan, 2012; Yang & Mousa, 2012). Bitter gourd (*Momordica charantia*) is a vegetable used in traditional medicine to treat T2D in Asia‐Pacific, African, and Caribbean countries. Its hypoglycemic activity has gained scientific support (Jia et al., 2017; Peter et al., 2019). Chromium is an essential mineral for carbohydrate and lipid metabolism. Benefits of trivalent (3^+^) chromium supplementation on T2D are recognized from well‐designed randomized clinical trials (Suksomboon et al., 2014; Wang & Cefalu, 2010).

There are apparently no reports of effects of functional foods on β‐cell dedifferentiation. This study aimed at testing whether an antidiabetic nutraceutical combination of red yeast rice, bitter gourd, and chromium was effective in preventing pancreatic β‐cell dedifferentiation. Mice with the *db*/*db* mutation in the C57BLKS background, a model of T2D with obesity, which are vulnerable to develop β‐cell failure upon compensation for insulin resistance, were used. These mice have lifelong hyperglycemia and hyperinsulinemia; over time, hyperglycemia worsens and blood insulin declines, with early‐stage β‐cell dedifferentiation apparent at 16–20 weeks (Gómez‐Banoy et al., 2019; Ishida et al., 2017; John et al., 2018; Sinha et al., 1979).

## 2 METHODS

### 2.1 Supplement preparation

This nutraceutical was composed of 60% red yeast rice (fermented by *Monascus purpureus*), 30% *Momordica charantia*, and 0.4% chromium nicotinate, with magnesium stearate and silicone dioxide as excipients (provided by Uni‐President Enterprises Corporation). Putative active principles monacolin K and chromium in this nutraceutical were 10 mg/g and 165 µg/g, respectively. A voucher specimen of *Momordica charantia* (UniPECCRI1807C) was deposited in the herbarium of Uni‐President Enterprises Corporation. The authenticity of *Monascus purpureus* was confirmed by the Institute of Microbiology, Chinese Academy of Sciences.

### 2.2 Animals and diets

Male BKS.Cg‐Dock7^m+/+^ Lepr^db^/JNarl (db/db) and wild‐type mice were purchased from the National Laboratory Animal Center of the National Applied Research Laboratories, Taipei, Taiwan, at 7 weeks of age. After acclimation for 1 week, db/db mice were allocated into 4 groups (*n* = 10 for each group), that is, DB, DB/L, DB/M, and DB/H, to receive a high‐fat diet (composition as described Chen et al. (2011)) containing 0%, 0.2%, 0.4%, or 1% nutraceutical, respectively. Wild‐type mice served as health control (C, *n* = 10) were fed a nonpurified standard diet (Altromin C1320, Fwusow Industry). All mice were kept in a room maintained at 23 ± 2°C, with a controlled 12‐hr light:dark cycle and free access to feed and drinking water. Feed intake and body weight were recorded every other day and weekly, respectively.

### 2.3 Sample collection

During the 8‐weeks dietary treatment, blood samples were collected from the tail vein at weeks 0, 2, 4, 6, and 8, after overnight fasting. An oral glucose tolerance test (OGTT) was performed at weeks 6. At weeks 7, mice were sequentially placed in a metabolic cage individually for 24 hr, recording water consumption and collecting urine. At the end point of the study, feed was withheld overnight and mice were killed by carbon dioxide asphyxiation. Portions of pancreas were fixed in 4% formaldehyde, pending histochemical analysis. Remaining pancreas were quick‐frozen in liquid nitrogen and stored at −80°C for subsequent extraction of RNA and protein. Serum was obtained, allowed to clot, centrifuged (3,000 × *g* for 10 min at 4°C), and frozen for subsequent determinations.

### 2.4 Oral glucose tolerance test

On the test days, after overnight food deprivation, blood was collected from the tail before (0 min), and at 30, 60, 90, and 120 min after oral gavage of a 2.5 M glucose solution (1.5 g/kg body weight). Whole‐blood glucose concentrations were measured using a MediSense Optium glucometer (Abbott Lab), and area under the curve (AUC) for blood glucose over the 2‐hr interval was calculated.

### 2.5 Measurement of biochemical indices

Glucose concentrations in serum and urine were measured enzymatically using GOD‐POP kits (Randox Lab). Enzyme‐linked immunosorbent assays were used to measure serum insulin (Millipore) and glucagon (Mercodia). Concentrations of albumin (PEG‐enhanced immunoturbidimetric method) and activity of serum GOT and GPT (IFCC method) were analyzed with an ADVIA Chemistry XPT system (ADVIA1800, SIEMENS).

### 2.6 Immunohistochemistry (IHC) analyses

Formaldehyde‐fixed tissues were dehydrated through a graded ethanol series, embedded in paraffin and 4‐µm cross‐sections were prepared. After deparaffinization and rehydration, sections were stained with hematoxylin and eosin and examined unblinded to treatment under a BX35 microscope (Olympus). Paraffin blocks were rehydrated with xylene, followed by decreasing concentrations of ethanol, permeabilized with 0.5% Triton X‐100 in PBS for 5 min, and blocked with 5% goat serum in PBS for 1 hr at room temperature. Primary antibodies used at a dilution of 1:50 in TBS containing 5% BSA were a guinea pig antibody against insulin (Abcam) and a rabbit antibody against glucagon (Cell Signaling) and NKX6.1 (Abcam). Alexa Fluor 488‐labeled goat anti‐guinea pig and Alexa Fluor 594‐labeled goat anti‐rabbit IgG antibodies (Abcam) were used as secondary antibodies. Images were acquired at 200× with a fluoromicroscope equipped with a SPOT RT color‐2000 digital camera (Diagnostic Instruments).

### 2.7 Immunoblotting

Tissues were homogenized in RIPA buffer containing 1% protease inhibitor cocktail and 1% phosphatase inhibitor cocktail (Sigma). Samples (40 µg of protein) were subjected to electrophoresis on 10% SDS gels, transferred to a polyvinylidene fluoride‐plus transfer membrane (Millipore), and immunoblotted. The primary antibodies, used at a dilution of 1:1,000 in TBS, were mouse antibody against β‐actin (Santa Cruz), and rabbit antibodies against NKX6.1, FOXO1, and ALDH1A3 (Abcam). The secondary antibody was HRP‐labeled donkey anti‐rabbit or anti‐mouse IgG antibody (Santa Cruz) at a dilution of 1:5,000 in TBST. Bound antibodies were detected using an enhanced chemiluminescence Western blotting kit (Millipore) and images quantified by densitometric analyses (Multimage Light Cabinet, Alpha Innotech Corporation).

### 2.8 RNA isolation and mRNA detection

Total RNA was extracted from pancreas using TRIzol reagent (Invitrogen), according to the manufacturer's instructions. Quality of extracted RNA was confirmed by a value of 2 for the 28S:18S ribosomal RNA ratio after ethidium bromide staining. Total RNA (1 µg) was reverse‐transcribed into first‐strand cDNA using 200 units of MMLV‐RT (Promega) in a total volume of 20 µl. For real‐time PCR, a SYBR system with self‐designed primers (Table S1) was used. Amplification using 40 cycles of 2 steps (95°C for 15 s and 60°C for 1 min) was done with an ABI Prism 7900HT sequence detection system.

### 2.9 Statistical analyses

Data are expressed as mean ± *SEM*. Comparisons among five groups were analyzed by 1‐way ANOVA and Duncan's multiple range test used to locate differences. If variances were not homogeneous, data were log‐transformed before statistical analyses. The general linear model procedure of SAS Version 9.4 was used for all statistical analyses (SAS Institute). Two‐tailed *p* < .05 denotes statistical significance.

## 3 RESULTS

**FIGURE 1:**
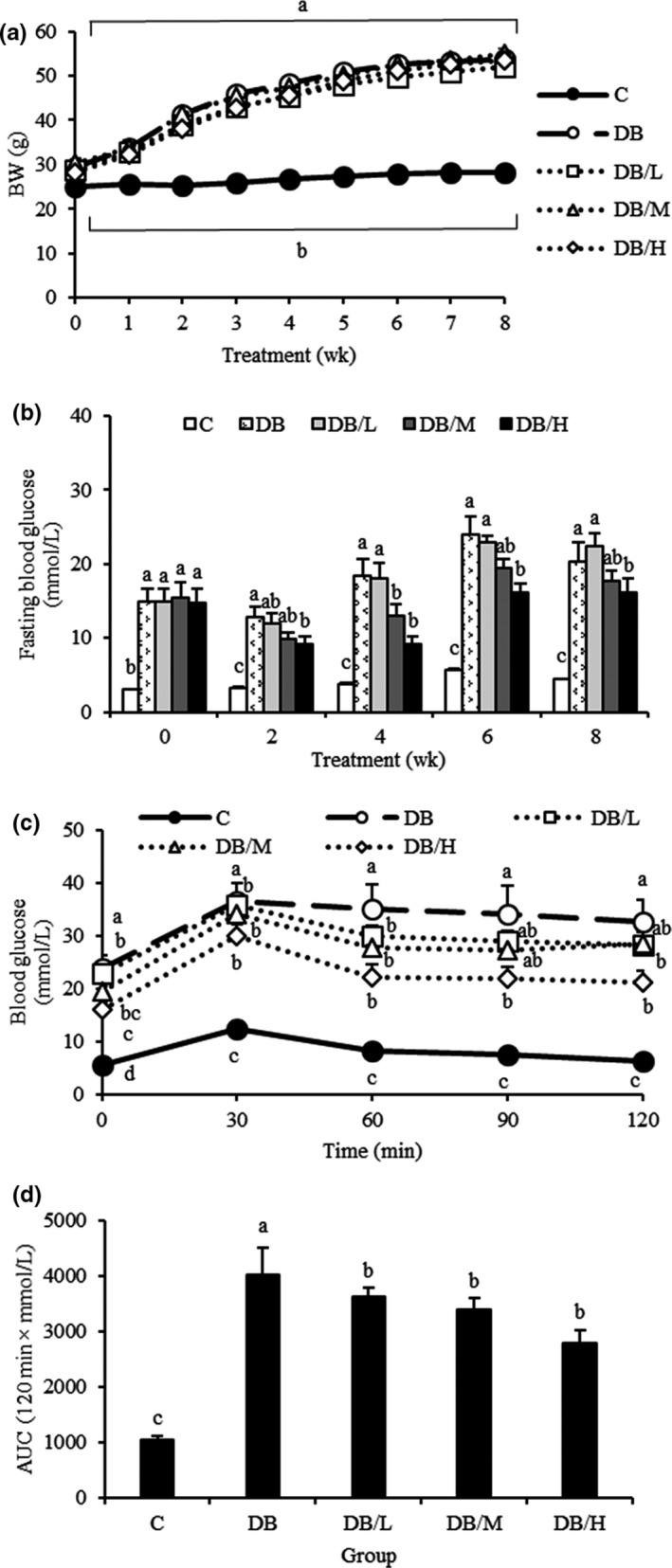
Growth curve (a), fasting blood glucose concentrations (b), oral glucose tolerance test blood glucose profile (c), and the AUC (d) for wild‐type (c group) and db/db mice fed 0%, 0.2%, 0.4%, or 1% doses of supplements (DB, DB/L, DB/M, and DB/H groups, respectively) for 8 weeks. Values are mean ± *SEM* (*n* = 8 or 10 for DB and other groups, respectively). ^a‐c^Within a time, means without a common letter differ, *p* < .05

In the DB group, 2 mice died during weeks 6–7; thus, their data were not included in the final analyses. Compared to the wild‐type mice (C group), db/db mice had obesity (Figure 1a), hyperglycemia (Figure 1b), hyperphagia, excessive drinking, polyuria, glucosuria, albuminuria, hyperinsulinemia (Table 1), and glucose intolerance (Figure 1c,d), as anticipated. Among db/db mice, though body weight and feed intake were not affected by the supplement, its antidiabetic function was apparent by ameliorating symptoms described above in a dose‐dependent manner. Compared to the DB group, fasting serum glucose concentrations were significantly reduced by DB/H at weeks 2–8 and by DB/M at weeks 4, although there was no reduction in DB/L (Figure 1b). Likewise, 24‐hr water consumption, urine output, urinary albumin excretion, and AUC of OGTT in DB/H group were significantly lower than those of DB and DB/L groups, whereas the DB/M group had intermediate values (Table 1 and Figure 1d). A supplement‐induced reduction in trend of glucosuria was also noted (Table 1). Elevated serum GOT and GPT in DB and DB/L groups (vs. C group) were significantly reduced in DB/M and DB/H groups, though still higher than the C group (Table 1).

**Table 1:** Diabetic parameters for wild‐type (C group) and db/db mice fed 0%, 0.2%, 0.4%, or 1% doses of supplements (DB, DB/L, DB/M, and DB/H groups, respectively) for 8 weeks^1^

Groups	C	DB	DB/L	DB/M	DB/H
Feed intake (kcal/d)	13.9 ± 0.7^b^	23.9 ± 0.6^a^	25.3 ± 0.6^a^	25.2 ± 0.9^a^	24.3 ± 0.7^ab^
Fasting serum insulin (pmol/L)	258 ± 130^c^	825 ± 172^b^	550 ± 115^b^	1324 ± 240^ab^	2220 ± 730^a^
24‐hr water intake (ml)	6.4 ± 1.1^b^	9.5 ± 0.5^a^	10.4 ± 0.7^a^	7.2 ± 0.5^ab^	5.1 ± 0.3^b^
24‐hr urine output (ml)	2.4 ± 0.3^b^	4.0 ± 0.4^a^	4.4 ± 0.5^a^	3.6 ± 0.3^ab^	2.8 ± 0.3^b^
Urine glucose (mmol/L)	1.3 ± 0.3^b^	709 ± 109^a^	730 ± 90^a^	670 ± 94^a^	479 ± 126^a^
Urinary albumin excretion (mmol/d)	0.00 ± 0.00^c^	1.40 ± 0.27^a^	2.41 ± 0.56^a^	0.99 ± 0.17^ab^	0.59 ± 0.16^b^
GOT (U/L)	471 ± 86^c^	1221 ± 187^a^	977 ± 368^ab^	626 ± 118^b^	667 ± 81^b^
GPT (U/L)	108 ± 22^c^	1043 ± 160^a^	754 ± 302^ab^	488 ± 90^b^	603 ± 75^b^

Note

a ^1^Values are mean ± *SEM* (*n* = 8 or 10 for DB and other groups, respectively). ^a‐c^Within a row, means without a common letter differ, *p* < .05.

**FIGURE 2:**
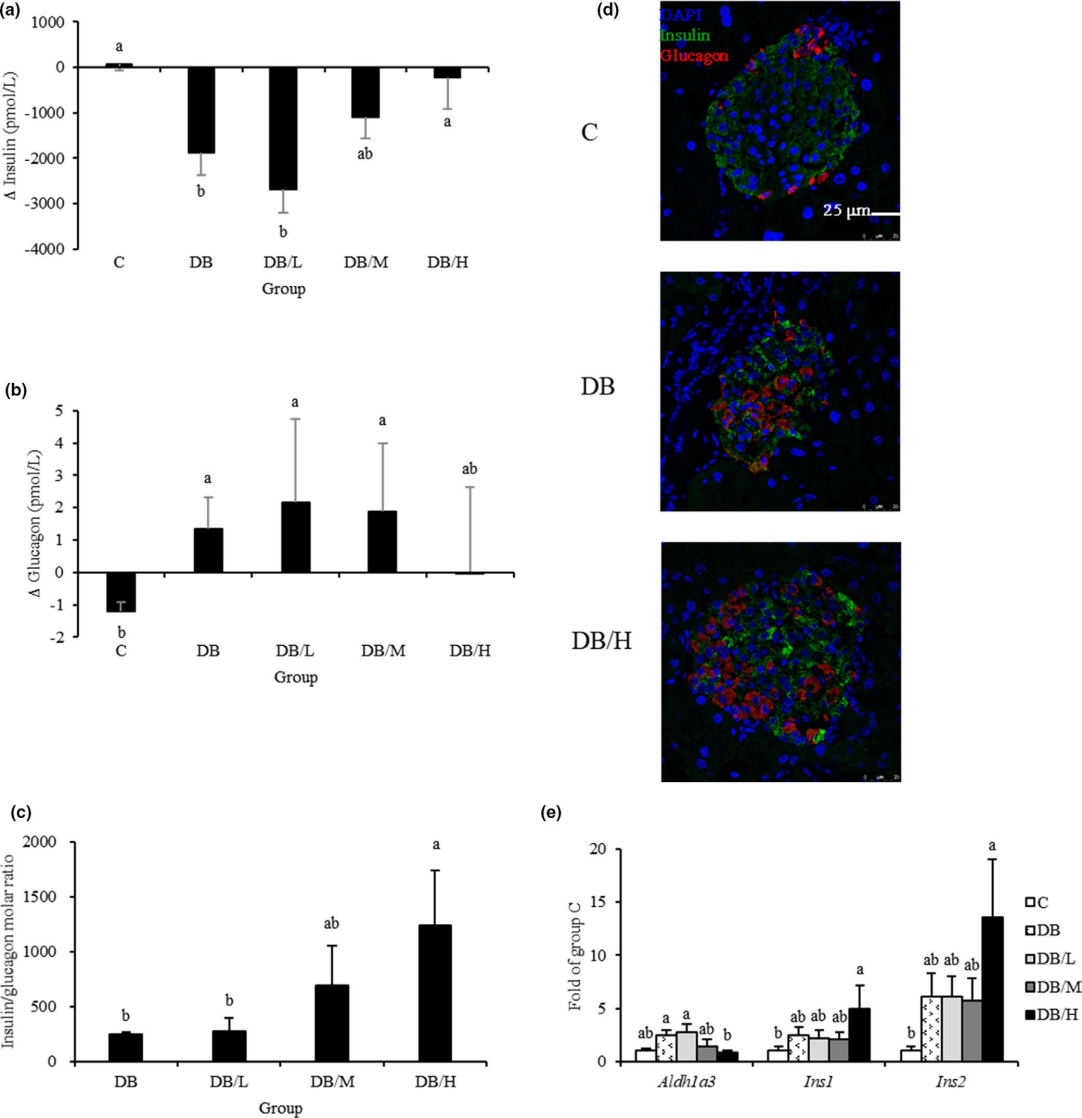
Changes in serum concentrations of insulin (a) and glucagon (b) during the study and serum insulin/glucagon molar ratio (c), pancreatic IHC staining for insulin and glucagon (d), and mRNA levels of markers associated with β‐cell (dys)function (e) for wild‐type (C group) and db/db mice fed 0%, 0.2%, 0.4%, or 1% doses of supplement (DB, DB/L, DB/M, and DB/H groups, respectively) for 8 weeks. In (d), representative pictures are shown. In (a–c and e), values are mean ± *SEM* (*n* = 8 or 10 for DB and other groups, respectively). ^a,b^Means without a common letter differ, *p* < .05

Though hyperinsulinemia is a hallmark of insulin resistance in db/db mice, at the end point, the highest fasting insulin concentration was in DB/H group, with significant differences from DB/L and DB groups (Table 1). To delineate underlying mechanisms, changes in serum concentrations of insulin and glucagon between weeks 0 and 8 were calculated. In contrast to almost no change across time for C and DB/H groups, there was a significant reduction in serum insulin in DB and DB/L groups, whereas a mild reduction occurred in DB/M group (Figure 2a). On the contrary, increasing serum glucagon concentrations across time were apparent in db/db mice, except for the DB/H group (Figure 2b). The molar ratio of insulin/glucagon was calculated for db/db mice; DB/H had significantly greater value than DB and DB/L groups, with an intermediate value for DB/M group (Figure 2c). Immunohistochemistry of pancreatic islets stained for insulin (β cells, green) and glucagon (α cells, red) confirmed mice in the DB group had less and weaker insulin staining than C and DB/H groups (Figure 2d). In line with the above, the DB/H group had the highest *Ins1* and *Ins2* expression levels of pancreatic mRNA (Figure 2e). Furthermore, *Aldh1a3* transcript, a β‐cell dedifferentiation marker, was significantly lower in DB/H than in DB or DB/L groups.

**FIGURE 3:**
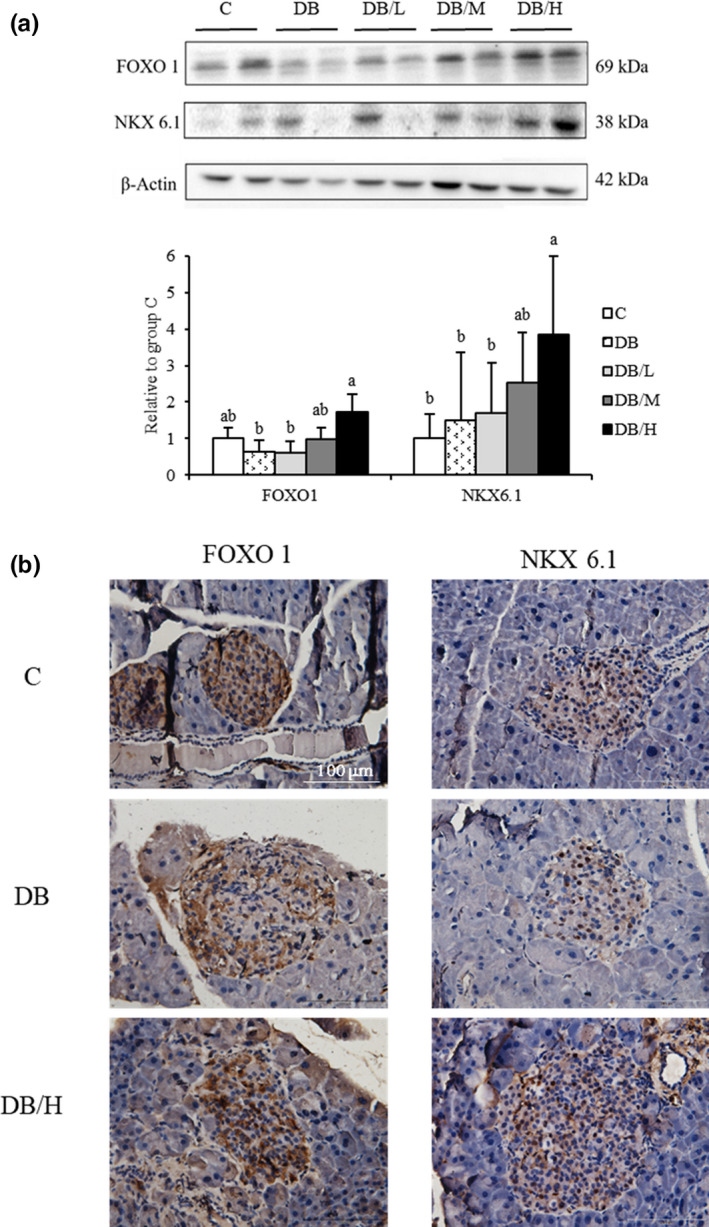
Western blotting (a) and IHC staining (b) for FOXO1 and NKX6.1 of pancreas from wild‐type (c group) and db/db mice fed 0%, 0.2%, 0.4%, or 1% doses of supplements (DB, DB/L, DB/M, and DB/H groups, respectively) for 8 weeks. Representative pictures are shown. In (a), values are mean ± *SEM* (*n* = 8 or 10 for DB and other groups, respectively). ^a,b^Means without a common letter differ, *p* < .05

FOXO1 and NKX6.1 protein levels in pancreatic homogenate were determined with Western blotting (Figure 3a). FOXO1 in the DB and DB/L groups was significantly lower than the DB/H group, whereas C and DB/M groups had intermediate values. Again, DB/H group had significantly greater levels of NKX6.1 than C, DB, and DB/L groups, with intermediate level for DB/M group. FOXO1 and NKX6.1 had higher intensity of staining (IHC) in islets of DB/H group than that of DB groups (Figure 3b).

**FIGURE 4:**
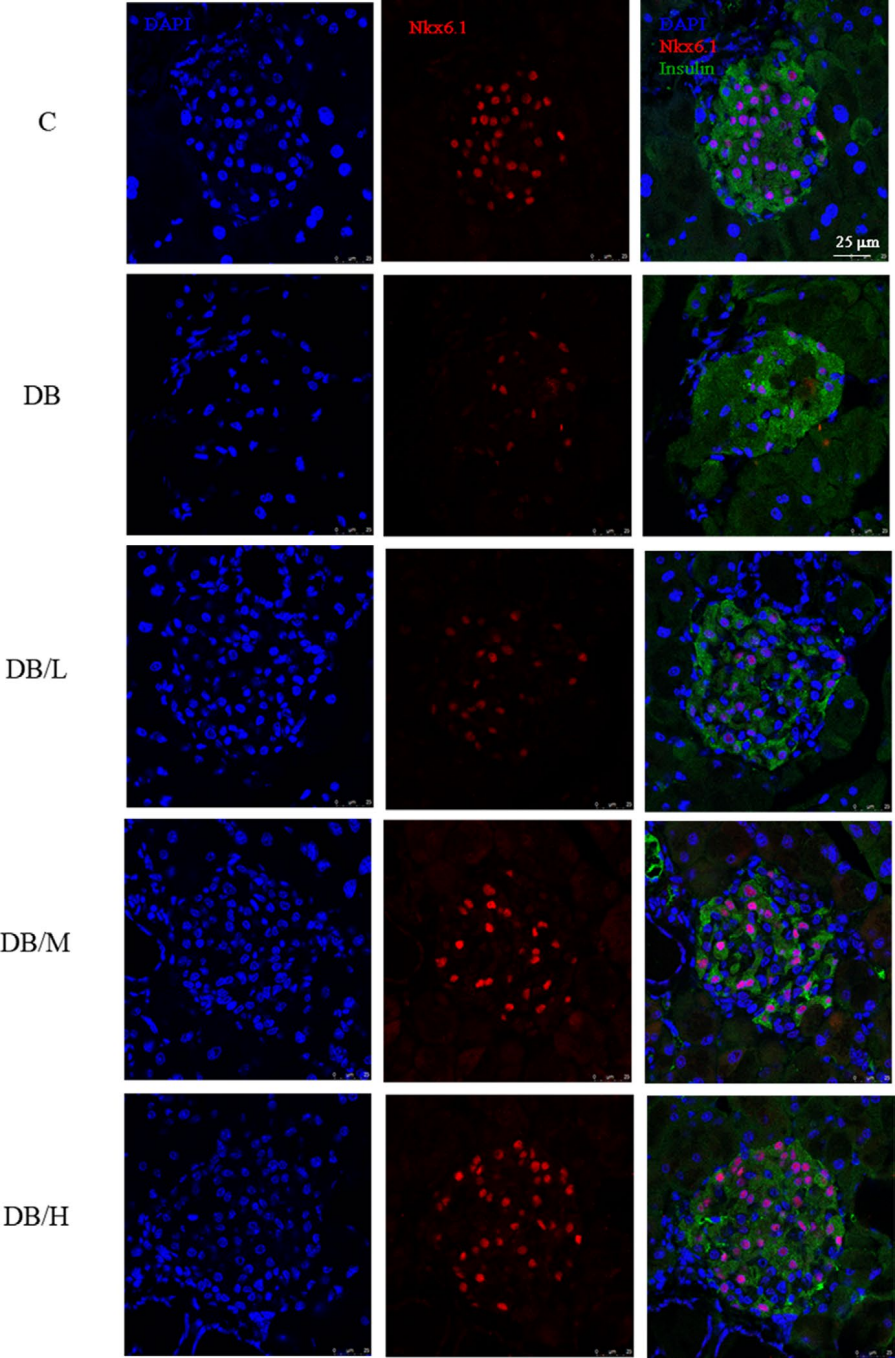
IHC staining for nuclear localization of NKX6.1 in islets of wild‐type (c group) and db/db mice fed 0%, 0.2%, 0.4%, or 1% doses of supplements (DB, DB/L, DB/M, and DB/H groups, respectively) for 8 weeks. Representative pictures are shown

To confirm nuclear and cytoplasmic distribution of NKX6.1 in β cells, pancreatic islets were stained with DAPI (nucleus, blue), NKX6.1 (red), and insulin (green) (Figure 4). Among insulin‐expressing cells, colocalization of DAPI and NKX6.1 (pink) was apparent in the C group, rarely detected in the DB and DB/L groups, but apparent in DB/M and D/H groups.

## 4 DISCUSSION

In this study, a combination of *Monascus*‐fermented rice, bitter gourd, and chromium nutraceutical had antidiabetes properties, as syndrome characteristics for T2D in db/db mice were dose‐dependently ameliorated by 8‐weeks supplementation. That this nutraceutical‐prevented β‐cell dedifferentiation was evidenced by reduced expression of dedifferentiation markers (or stem cell marker) *Aldh1a3*, with increased expression of β‐cell‐enriched transcription factors (FOXO1, NKX6.1) in pancreatic islets, as compared to diabetic control. Accordingly, by conserving more β cells with secretary function, decreases in serum insulin concentrations or insulin/glucagon ratio observed in db/db mice at 16 weeks of age were attenuated or absent in supplementation groups DB/M and DB/H. Again, these changes were dose‐dependent. It should be noted that 2 mice with severe hyperglycemia in the DB group died during the study period, which could have resulted in an underestimate of disease parameters in the diabetic control group, thus explaining no difference between DB and DB/L groups.

FOXO1 is required to cope with metabolic stress by maintaining β‐cell identity and function (Talchai et al., 2012). Downregulation of FOXO1 may be caused by chronic hyperglycemia‐induced oxidative stress; consequently, β‐cell fate cannot be maintained, due to insufficient β‐cell transcription factors, PDX1, MAFA, and NKX6.1, all of which are FOXO1‐dependent (Kitamura et al., 2005). NKX6.1, a transcriptional repressor that is tightly restricted to β‐cell nuclei in adult islets, suppresses glucagon expression (Watada et al., 2000). Based on IHC, the nuclear localization of NKX6.1 was diminished in diabetic islets, but dose‐dependently increased by the supplementation, in concert with changes in serum insulin and glucagon concentrations and their IHC staining (Figure 2). Reciprocal changes in insulin and glucagon staining in islets of groups DB and C were evidence that dedifferentiated β cells underwent conversion into α cells. Reductions in insulin/glucagon ratios and hyperglucagonemia occur in human T2D (Dunning & Gerich, 2007).

Though many diabetic treatments were reported to improve β‐cell functions, few have been tested for preventing or reversing β‐cell dedifferentiation. In comparisons of pair feeding (calorie restriction), insulin, SGLT2‐inhibitor phloridzin, and insulin sensitizer rosiglitazone in db/db mice, only pair feeding successfully suppressed markers of β‐cell dedifferentiation (Ishida et al., 2017), suggesting either alleviation of hyperglycemia or reduction of the secretory workload on β cells will not reverse β‐cell dedifferentiation. Results from that study supported conclusions that lifestyle modifications are superior to pharmacological treatments, as frequently demonstrated in Diabetes Prevention Trials. However, another study suggested any therapeutic approach that reduced insulin secretion demand and enabled β‐cell rest, irrespective of whether it alleviated insulin resistance or improved glucose homeostasis, preserved functional β cells (Boland et al., 2019). Furthermore, a combination of any 2 treatments (GLP‐1 analog liraglutide, SGLT‐2 inhibitor dapagliflozin or insulin sensitizer thiazolidinedione) had better effects than a single treatment (Boland et al., 2019). Restoring β‐cell function by allowing β‐cell rest was also supported by temporary intensive insulin therapy in early diagnosed T2D patients (Weng et al., 2008). Moreover, islets isolated from db/db mice, if removed from their in vivo milieu (hyperglycemia and insulin resistance) and cultured in euglycemia, rapidly recovered glucose‐stimulated insulin secretion capacity (Alarcon et al., 2016).

Compounds with antidiabetic activity in *Monascuc‐*fermented products mainly focus on monacolins, γ‐aminobutyric acid (GABA), monascin, and ankaflavin, working through mechanisms such as anti‐inflammation, anti‐oxidation, PPARγ activation, and augmentation in β‐cell insulin secretion by increasing acetylcholine release from nerve terminals (Chen & Liu, 2006; Hsu et al., 2013; Lin et al., 2017; Shi & Pan, 2012). In accordance with preventing dedifferentiation of β cells and their conversion into α cells as suggested in this study, an earlier study reported phosphoenolpyruvate carboxykinase (a rate‐limiting enzyme for gluconeogenesis in response to glucagon and insulin) was downregulated in the liver of diabetic rats treated with red yeast rice (Chang et al., 2006). In addition, induction of hepatic FOXO1 or DAF‐16/FOXO was also documented in monascin‐treated diabetic rats and *Caenorhabditis elegans* (Shi et al., 2012; Shi & Pan, 2012).

The antidiabetic activity of bitter gourd has been intensively investigated. Functional components such as charantin, vicin, polypeptide P (P‐insulin), momordicoside Q, R, S and T, conjugated linolenic acid, and some cucurbitane‐type triterpenoids (saponins), present in seeds or fruits, with various mechanisms of action, including insulin‐like effect, PPARγ activation (insulin sensitizer), α‐glucosidase inhibitor, secretagogues, etc., have been proposed (Alam et al., 2015; Joseph & Jini, 2013; Oh, 2015; Wang et al., 2017). Recovery/renewal of partially destroyed pancreatic β cells was also documented in earlier reports of streptozotocin‐treated adult and neonatal rats supplemented with *Momordica charantia* (Abdollahi et al., 2011; Ahmed et al., 1998). Whether this was associated with prevention of β‐cell dedifferentiation remains unknown.

The antidiabetic actions of chromium were proposed as enhancing insulin binding, insulin receptor number, or linking with chromodulin to increase receptor signaling (Wang & Cefalu, 2010). However, we are not aware of any reports regarding improving β‐cell function.

Though clinical studies are scarce for *Monascus*‐fermented products, based on meta‐analysis of randomized clinical trials, when bitter gourd or chromium are given independently, doses of 2–6 g/d (Peter et al., 2019) and 200–1000 µg/d (Suksomboon et al., 2014), respectively, are recommended to achieve glycemic control. The medium (DB/M) or high (DB/H) dose used in this mouse‐feeding trial approached the minimum requirement for human efficacy. Considering experience from Boland et al. (2019), we inferred the protective effect of this nutraceutical on β‐cell dedifferentiation was due to multiple ingredients that acted synergistically through various mechanisms, thus reducing metabolic load and effectively “resting” β cells.

There is a long journey from functional impairment of β cells to their demise. Curbing β‐cell dedifferentiation (by relieving metabolic load or allowing β‐cell rest) as early as possible is a good entry point to curtail disease progress by conserving more β cells with functions. In a model of rhesus monkeys fed a high‐fat/high‐sugar diet (Westernized diet), Fiori et al. demonstrated β‐cell dedifferentiation occurred early, even before hyperglycemia was present (prediabetes). Resveratrol activated Sirtuin 1 and unexpectedly prevented β‐cell dedifferentiation, rather than improving insulin resistance associated with a Westernized diet (Fiori et al., 2013). The current study provided the impetus to develop various combinations of nutraceuticals that target inhibition of β‐cell dedifferentiation; in addition to adjuncts for diabetes treatment (e.g., lowering medication dose), more importantly they may prevent or delay progress of prediabetes into diabetes.

## 5 CONCLUSION

A nutraceutical combination of red yeast rice, bitter gourd, and chromium could be used as a dietary supplement for T2D, as it prevented β‐cell dedifferentiation in db/db mice, thus slowing the disease progress by conserving more β cells with secretary function.

## CONFLICT OF INTEREST

The authors declare that they have no conflict of interest.

## ETHICAL APPROVAL

This study was approved by the Institutional Animal Care and Use Committee of China Medical University (CMUIACUC‐2018‐279‐1). All procedures performed in studies involving animals were in accordance with the ethical standards and practices of the institution where the study was conducted.

## Supporting information

Table S1

## Data Availability

The data that support the findings of this study are available from the corresponding author upon reasonable request.
