# Supplementary material for: An antidiabetic nutraceutical combination of red yeast rice (Monascus purpureus), bitter gourd (Momordica charantia), and chromium alleviates dedifferentiation of pancreatic β cells in db/db mice
Source: Food Sci Nutr. 2020 Oct 25;8(12):6718–26. doi: 10.1002/fsn3.1966 (PMC7723183; doi:10.1002/fsn3.1966)
Supplement: Supplementary file 1 — Table S1 [file FSN3-8-6718-s001.docx]

Table S1 Gene names and sequences of PCR primers

| Gene | Encoding protein | Accession no. | Primer |
| --- | --- | --- | --- |
| *Aldh1a3* | Aldehyde dehydrogenase family 1, subfamily A3 | NM_053080.3 | F: ACTGGAGCTAGGAGGCAAGAAC  R: GTAGACCTGCTCTTCCACGAAC |
| *Ins1* | Insulin-1 | NM_008386.4 | F: CCAGCTATAATCAGAGACCA  R: GGGCCTTAGTTGCAGTAGTT |
| *Ins2* | Insulin-2 | NM_001185084.2 | F: AGCCCTAAGTGATCCGCTACAA  R: AGTTGCAGTAGTTCTCCAGCTG |
